# Impact of vessel morphology on CT-derived fractional-flow-reserve in non-obstructive coronary artery disease in right coronary artery

**DOI:** 10.1007/s00330-023-09972-8

**Published:** 2023-09-01

**Authors:** Toshimitsu Tsugu, Kaoru Tanaka, Dries Belsack, Yuji Nagatomo, Mayuko Tsugu, Jean-François Argacha, Bernard Cosyns, Nico Buls, Michel De Maeseneer, Johan De Mey

**Affiliations:** 1 Department of Radiology, Universitair Ziekenhuis Brussel, Laarbeeklaan 101, 1090 Jette, Brussels, Belgium; 2 Department of Cardiology, National Defense Medical College Hospital, Tokorozawa, Japan; 3 Cardiology, Centrum Voor Hart- en Vaatziekten, Universitair Ziekenhuis Brussel, Brussels, Belgium

**Keywords:** Coronary artery disease, Computed tomography angiography, Ischemia

## Abstract

**Objectives:**

Computed tomography (CT)–derived fractional flow reserve (FFR_CT_) decreases continuously from proximal to distal segments of the vessel due to the influence of various factors even in non-obstructive coronary artery disease (NOCAD). It is known that FFR_CT_ is dependent on vessel-length, but the relationship with other vessel morphologies remains to be explained.

**Purpose:**

To investigate morphological aspects of the vessels that influence FFR_CT_ in NOCAD in the right coronary artery (RCA).

**Methods:**

A total of 443 patients who underwent both FFR_CT_ and invasive coronary angiography, with < 50% RCA stenosis, were evaluated. Enrolled RCA vessels were classified into two groups according to distal FFR_CT_: FFR_CT_ ≤ 0.80 (*n* = 60) and FFR_CT_ > 0.80 (*n* = 383). Vessel morphology (vessel length, lumen diameter, lumen volume, and plaque volume) and left-ventricular mass were assessed. The ratio of lumen volume and vessel length was defined as V/L ratio.

**Results:**

Whereas vessel-length was almost the same between FFR_CT_ ≤ 0.80 and > 0.80, lumen volume and V/L ratio were significantly lower in FFR_CT_ ≤ 0.80. Distal FFR_CT_ correlated with plaque-related parameters (low-attenuation plaque, intermediate-attenuation plaque, and calcified plaque) and vessel-related parameters (proximal and distal vessel diameter, vessel length, lumen volume, and V/L ratio). Among all vessel-related parameters, V/L ratio showed the highest correlation with distal FFR_CT_ (*r* = 0.61, *p* < 0.0001). Multivariable analysis showed that calcified plaque volume was the strongest predictor of distal FFR_CT_, followed by V/L ratio (β-coefficient = 0.48, *p* = 0.03). V/L ratio was the strongest predictor of a distal FFR_CT_ ≤ 0.80 (cut-off 8.1 mm^3^/mm, AUC 0.88, sensitivity 90.0%, specificity 76.7%, 95% CI 0.84–0.93, *p* < 0.0001).

**Conclusions:**

Our study suggests that V/L ratio can be a measure to predict subclinical coronary perfusion disturbance.

**Clinical relevance statement:**

A novel marker of the ratio of lumen volume to vessel length (V/L ratio) is the strongest predictor of a distal CT-derived fractional flow reserve (FFR_CT_) and may have the potential to improve the diagnostic accuracy of FFR_CT_.

**Key Points:**

*• Physiological FFR*_*CT*_ *decline depends not only on vessel length but also on the lumen volume in non-obstructive coronary artery disease in the right coronary artery.*

*• FFR*_*CT*_ *correlates with plaque-related parameters (low-attenuation plaque, intermediate-attenuation plaque, and calcified plaque) and vessel-related parameters (proximal and distal vessel diameter, vessel length, lumen volume, and V/L ratio).*

*• Of vessel-related parameters, V/L ratio is the strongest predictor of a distal FFR*_*CT*_ *and an optimal cut-off value of 8.1 mm* ^*3*^ */mm.*

**Supplementary information:**

The online version contains supplementary material available at 10.1007/s00330-023-09972-8.

## Introduction

Computed tomography (CT)–derived fractional flow reserve (FFR_CT_) is a feasible tool for assessing the hemodynamic significance of coronary stenosis non-invasively with a high diagnostic accuracy of 81.9% [1]. The implementation of FFR_CT_ can change treatment strategies and improve diagnostic efficiency and effectiveness in patients with coronary artery disease (CAD) [2]. Even in non-obstructive CAD (NOCAD), FFR_CT_ gradually decreases from the proximal to the distal segments of the vessel (physiological FFR_CT_ decline) [3, 4]. FFR_CT_ is influenced by various factors such as vessel length [3, 4], bifurcation angle [5, 6], plaque burden [3], left ventricular mass [7], and collateral circulation [8]. FFR_CT_ is modified by multiple variables, resulting in 37% of vessels presenting FFR_CT_ of ≤ 0.80 even with normal coronary arteries [9]. Vessel length plays an important role in physiological FFR_CT_ decline [3, 4]. It is empirically proven that differing FFR_CT_ changes occur depending on the lumen volume even with the same vessel length. The lumen cross-sectional area decreases towards the distal segments [10], resulting in vessel tapering. The lumen cross-sectional area correlates with FFR_CT_ [11]. Furthermore, with a higher dose of nitroglycerin as a vasodilator agent, lumen volume increases, resulting in FFR_CT_ elevation at the distal vessel segments [12]. Hence, physiological FFR_CT_ decline may be related to lumen volume [9]. The relationship between vessel length and lumen volume and FFR_CT_ remains to be explained. The present study aimed to investigate morphological aspects of the vessels and to identify predictive factors forphysiological FFR_CT_ decline in NOCAD in the right coronary artery (RCA).

## Methods

### Patient population

**Fig. 1:**
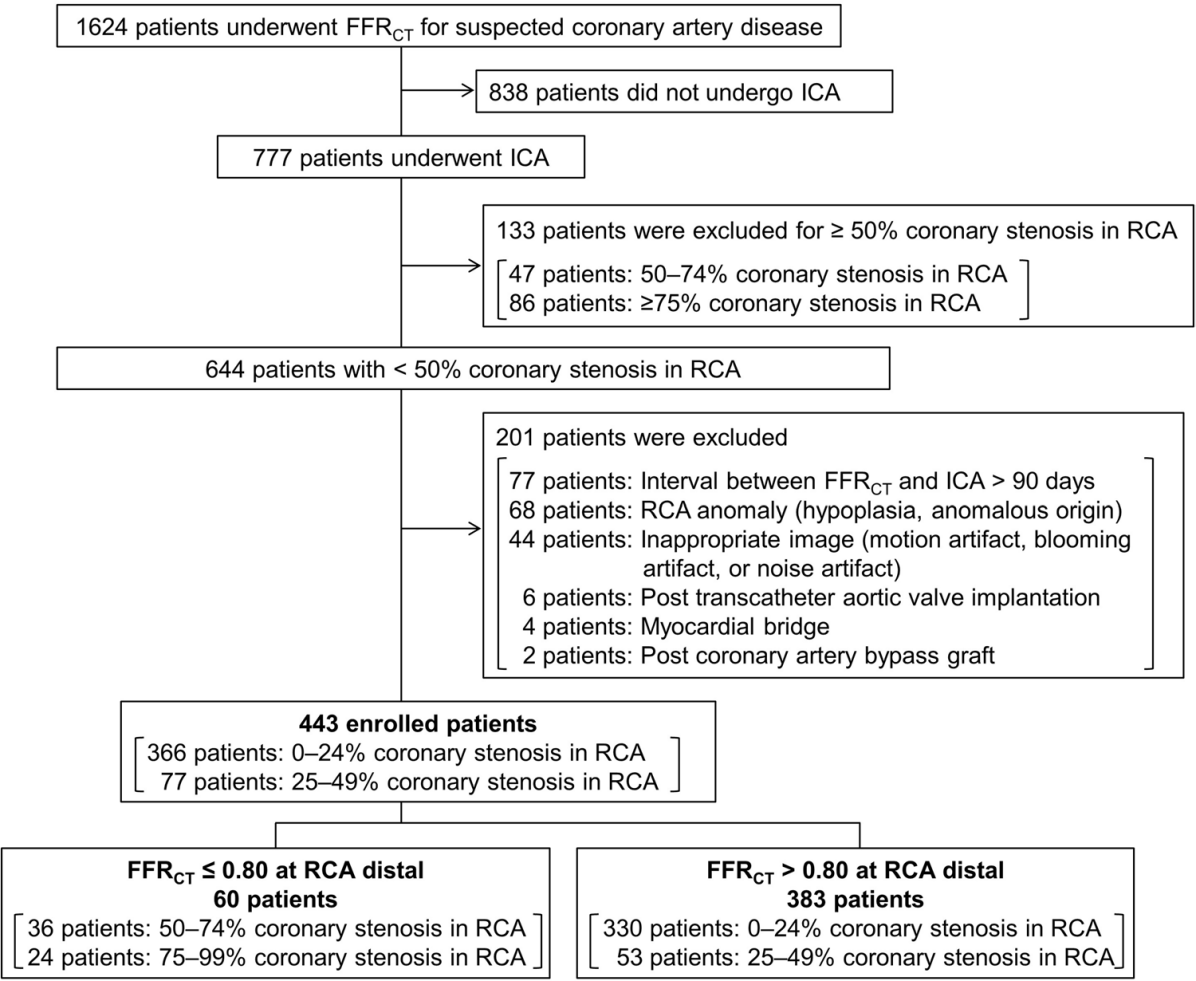
Patient selection flow diagram showing patient flow in the study. CTA, computed tomography angiography; ICA, invasive coronary angiography; RCA, right coronary artery

A total of 1624 outpatients with suspected CAD and who had a CT angiography (CTA) with FFR_CT_ analysis examined at the Universitair Ziekenhuis Brussel between January 2017 and January 2023 were included in the study. A retrospective cohort study was performed. Approval from the ethical board at the Universitair Ziekenhuis Brussel was obtained with protocol number B.U.N. 143202000302. Inclusion criteria were NOCAD on ICA at the RCA. The presence of coronary stenosis in the left coronary artery was irrelevant. NOCAD was defined as vessels with < 50% coronary stenosis or luminal irregularities. The severity of coronary stenosis on CTA was determined as 0–2 according to CAD reporting and data system (CAD-RADS) classification [13] by experts in cardiac radiology imaging (T.T., and K.T.). The severity of coronary stenosis was assessed visually by experienced interventional cardiologists (JF.A. and C.B.). A total of 838 patients who had not undergone ICA and 133 patients with ≥ 50% coronary stenosis were excluded from the study. Although not included in the current study, patients with heart failure and old myocardial infarction were excluded. The following categories of patients were also excluded from the study: time interval between FFR_CT_ and ICA > 90 days (*n* = 77), RCA anomaly (hypoplasia, anomalous origin) (*n* = 68), inappropriate image quality (motion artifact, blooming artifact, or noise artifact) (*n* = 44), post transcatheter aortic valve implantation (*n* = 6), myocardial bridge (*n* = 4), post coronary artery bypass graft (*n* = 2). As a result, 443 patients were enrolled and divided into two groups according to RCA distal vessel FFR_CT_: FFR_CT_ ≤ 0.80 (*n* = 60) and FFR_CT_ > 0.80 (*n* = 383). The patient selection flowchart is presented in Fig. 1.

### Coronary CT angiography

All coronary CTA scans were acquired with a 320 slice GE Revolution scanner (GE Healthcar) enabling to image the heart in one heartbeat. This protocol used a single-rotation axial scan with prospective gating, 16-cm detector coverage, noise index 30 (noise level fixed over all kVp levels and body sizes), and gantry rotation time of 0.28 s. Tube potential ranged between 70 and 120 kV depending on body size. Iodinated contrast agent was administered with Iopromide 370 (Bayer Schering Pharma) using the bolus tracking method at a rate of 5.0 ml/s depending on body size. The volume of iodinated contrast injected was individualised according to body size (50–80 mL). Beta-blockers were administered when necessary to obtain a target heart rate of < 60 beats/min. According to guidelines [14], sublingual nitrates (two sprays of 0.8 mg) were administered 5 min prior to CT scanning in all patients. The images were reconstructed at 75 ± 10% of the R-R interval.

### FFR_CT_

**Fig. 2:**
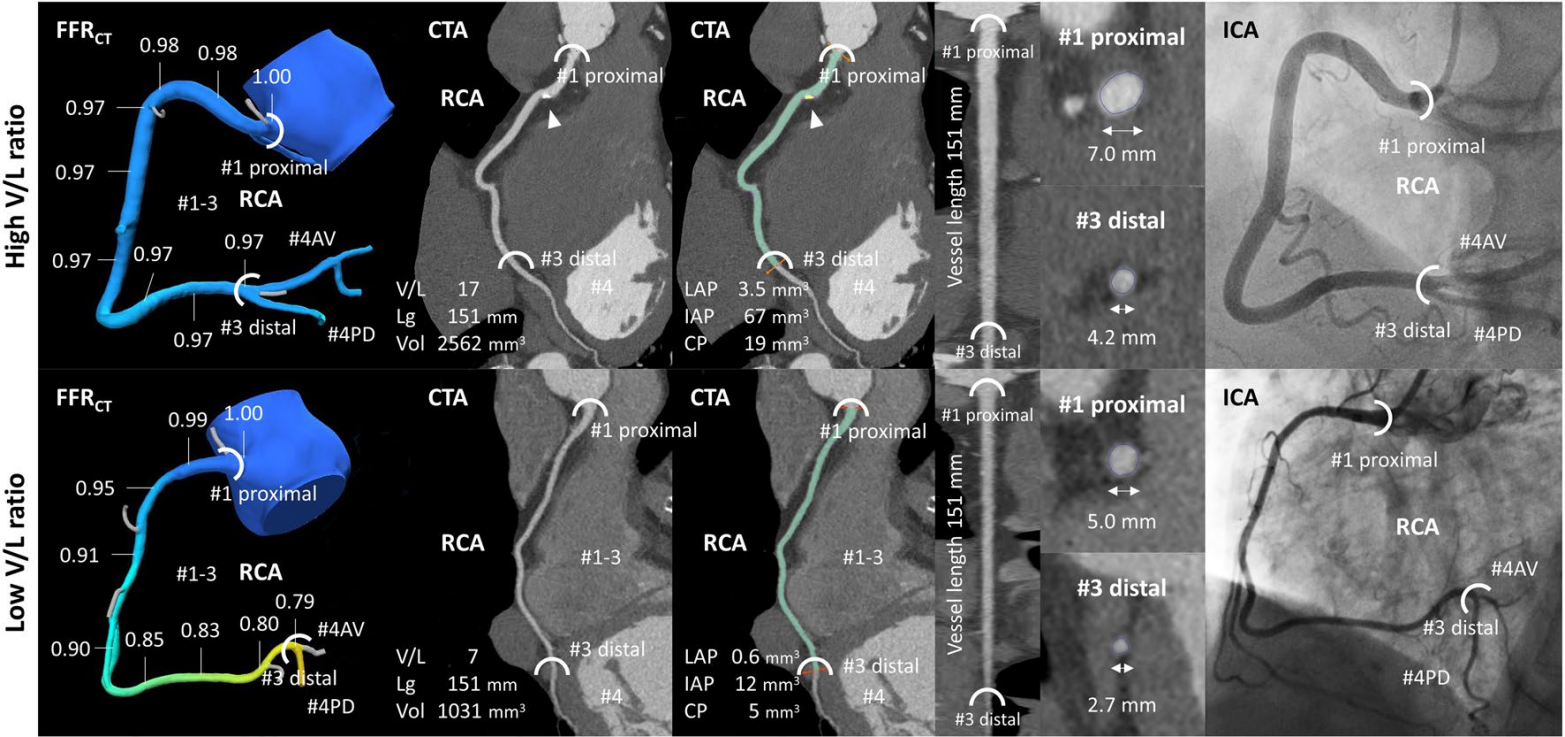
Representative cases of high V/L ratio (top panel) and low V/L ratio (bottom panel). Analysis range is from #1 proximal to #3 distal. Arrowhead (CP). AV, atrioventricular nodal; CP, calcified plaque; IAP, intermediate-attenuation plaque; LAP, low-attenuation plaque; Lg, vessel length; PD, posterior descending; Vol, lumen volume

FFR_CT_ was analysed by HeartFlow Inc. Computational fluid dynamics and blood flow simulations were performed to calculate the FFR_CT_ at any arbitrary point in the coronary artery. The RCA was classified into 4 segments (#1–#4) based on the American Heart Association classification (AHA) [15]. To avoid the effects of turbulence due to branching, FFR_CT_ values were measured from RCA ostium (#1) to the bifurcation (#3 distal end) of the atrioventricular nodal branch (#4AV) and posterior descending branch (#4PD) (Fig. 2). Moreover, each segment was divided into three categories (proximal, middle, and distal). The magnitude of change in FFR_CT_ (ΔFFR_CT_) was measured from the proximal to the distal at the RCA. A positive FFR_CT_ was defined as a value ≤ 0.80 in accordance with previously published literature [16–19].

### Coronary artery morphology and composition

Vessel diameter, vessel length, lumen volume, and composition of each vessel were measured using GE AW server 3.2 software (GE Healthcare). The ratio of lumen volume and vessel length was defined as V/L ratio (mm^3^/mm). Plaque characterisation and vessel morphology measurements were performed semi-automatically with Color Code Plaque (GE Healthcar). Vessel constituents were characterised based on Hounsfield units (HU) into low-attenuation plaque (LAP) (< 30 HU), intermediate-attenuation plaque (IAP) (30–150 HU), and calcified plaque (CP) (> 150 HU) [20, 21].

### Invasive coronary angiography

All ICA images were evaluated by Philips Azurion with ClarityIQ (Philips Healthcare). Iodixanol 320 (GE Healthcare) was administered as an iodinated contrast agent.

### Statistical analysis

Continuous variables were expressed as mean ± standard deviation (SD). The 95% confidence interval was calculated as ± 1.96 SDs from the mean. Two groups (FFR_CT_ > 0.80 and ≤ 0.80) comparisons were performed with paired t test or 1-way analysis of variance for means if the data were normally distributed or with Mann–Whitney *U* test or Kruskal–Wallis test if the data were not normally distributed. Fisher’s or *χ*^2^ test was used to analyse the categorical data. Associations between distal FFR_CT_, ΔFFR_CT_, and cardiac parameters were assessed by Pearson correlation analysis. Multivariable linear regression analyses were performed to examine the independent correlates between distal FFR_CT_, ΔFFR_CT_, and cardiac parameters. Receiver operating characteristic curves and their area under the curve were estimated based on Mann–Whitney U test, and the null hypothesis on the area under the curve C (H0: area under the curve = 0.5) was tested. Receiver operating characteristics curves were generated to determine the cut-off value of the highest diagnostic performance of an FFR_CT_ ≤ 0.80 at the distal vessel. Intra-observer (T.T.) and inter-observer (T.T. and K.T.) agreement was assessed in 15 randomly selected subjects using Bland–Altman analyses. *p* < 0.05 was considered statistically significant. All statistical analyses were performed with JMP 11.0 statistical software (SAS Institute) or R version 4.4.2 (R Foundation for Statistical Computing).

## Results

### Physical, CT acquisition condition, FFR_CT_, and vessel characteristics

**Fig. 3:**
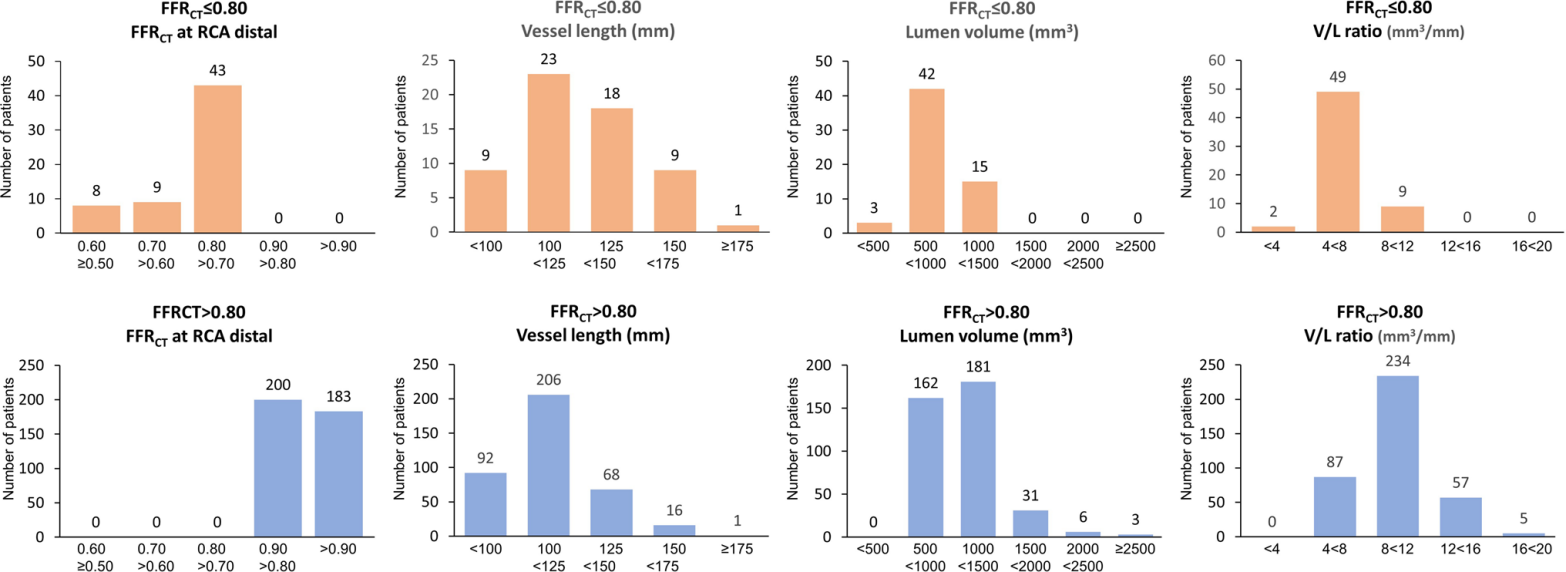
Distribution of FFR_CT_ and vessel morphology

**Fig. 4:**
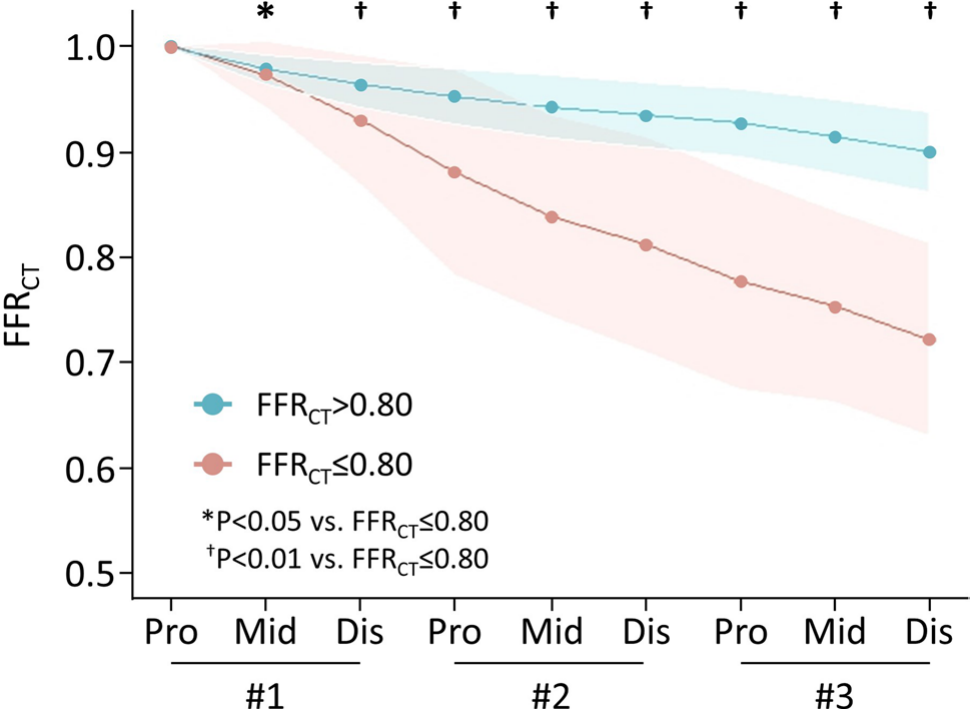
Changes in FFR_CT_ between FFR_CT_ ≤ 0.80 and > 0.80

Among 443 patients, 383 patients (86%) had FFR_CT_ > 0.80, whereas 60 patients (14%) showed a positive FFR_CT_ even in NOCAD in the RCA (Fig. 3). Table 1 summarises the physical, CT acquisition condition, FFR_CT_, vessel, and myocardial characteristics in the RCA in our study population. Physical characteristics did not differ between FFR_CT_ ≤ 0.80 and > 0.80. The time phase in the cardiac cycle during CT images could be acquired at the mid-diastolic phase in all patients. Proximal FFR_CT_ was identical between FFR_CT_ ≤ 0.80 and > 0.80, but distal FFR_CT_ (0.72 ± 0.09 vs. 0.90 ± 0.04, *p* < 0.0001) and ΔFFR_CT_ (0.28 ± 0.09 vs. 0.10 ± 0.04, *p* < 0.0001) were significantly lower in FFR_CT_ ≤ 0.80 than in > 0.80 (Fig. 4, Table 1, and Supplementary Table 1). Vessel length did not differ between FFR_CT_ ≤ 0.80 and > 0.80 (118.3 ± 22.3 vs. 113.4 ± 17.4 mm, *p* = 0.11). Proximal vessel diameter, distal vessel diameter, lumen volume (825.7 ± 255.0 vs. 1113.0 ± 337.9 mm^3^, *p* < 0.0001), and V/L ratio (6.5 ± 1.6 vs. 9.9 ± 2.4 mm^3^/mm, *p* < 0.0001) were significantly lower in FFR_CT_ ≤ 0.80 than in > 0.80. Vessel length was almost the same, but vessel morphology (proximal vessel diameter, distal vessel diameter, lumen volume, and V/L ratio) was significantly lower in FFR_CT_ ≤ 0.80. Distal FFR_CT_ was lower in ≤ FFR_CT_ > 0.80. LAP volume was almost the same comparing FFR_CT_ ≤ 0.80 and > 0.80, but IAP and CP volume were higher in FFR_CT_ ≤ 0.80 (Table 1).

**Table 1:** Physical, CT acquisition condition, FFR_CT_, vessel, and myocardial characteristics in the right coronary artery

	All *n* = 443	FFR_CT_ ≤ 0.80 *n* = 60	FFR_CT_ > 0.80 *n* = 383
Physical characteristics			
Age (years)	67.2 ± 9.9	69.3 ± 7.8	66.9 ± 10.2
Men, *n* (%)	304 (69%)	36 (60%)	310 (81%)
Height (cm)	172.6 ± 9.5	171.4 ± 11.2	172.8 ± 9.2
Body weight (kg)	81.0 ± 15.2	77.7 ± 17.2	81.5 ± 14.8
Body surface area (m^2^)	1.9 ± 0.2	1.9 ± 0.3	1.9 ± 0.2
Body mass index (kg/m^2^)	27.1 ± 4.3	26.2 ± 4.3	27.2 ± 4.3
Hypertension, *n* (%)	92 (51%)	11 (58%)	81 (51%)
Dislipidemia, *n* (%)	73 (41%)	8 (42%)	65 (41%)
Diabetes, *n* (%)	33 (19%)	5 (26%)	28 (18%)
Current smoking, *n* (%)	57 (32%)	7 (35%)	50 (31%)
CT images acquisition condition			
Time phase in the cardiac cycle during CT imaging	75.0 ± 5.4	74.7 ± 4.6	75.1 ± 5.5
Heart rate (beats/min)	60.3 ± 8.6	62.5 ± 9.2	59.9 ± 8.5
Atrial fibrillation, *n* (%)	46 (10%)	7 (12%)	39 (10%)
Systolic blood pressure (mmHg)	143.8 ± 19.8	147.2 ± 10.9	142.3 ± 19.3
Diastolic blood pressure (mmHg)	83.1 ± 12.1	87.8 ± 12.8	82.4 ± 11.9
Interval between FFR_CT_ and invasive coronary angiography (days)	22.0 ± 18.4	21.0 ± 15.0	22.1 ± 18.8
FFR_CT_ characteristics			
Proximal FFR_CT_	1.00 ± 0.00	1.00 ± 0.00	1.00 ± 0.00
Distal FFR_CT_	0.88 ± 0.08	0.72 ± 0.09^†^	0.90 ± 0.04
ΔFFR_CT_	0.12 ± 0.08	0.28 ± 0.09^†^	0.10 ± 0.04
Vessel morphology			
Vessel length (mm)	114.0 ± 18.1	118.3 ± 22.3	113.4 ± 17.4
Lumen volume (mm^3^)	1074.0 ± 342.3	825.7 ± 255.0^†^	1113.0 ± 337.9
Proximal vessel diameter (mm)	4.5 ± 1.0	3.9 ± 0.8^†^	4.6 ± 1.0
Distal vessel diameter (mm)	2.8 ± 0.6	2.4 ± 0.5^†^	2.9 ± 0.6
V/L ratio (mm^3^/mm)	9.4 ± 2.6	6.5 ± 1.6^†^	9.9 ± 2.4
LAP volume (mm^3^)	15.6 ± 18.5	18.6 ± 18.0	15.1 ± 18.6
IAP volume (mm^3^)	156.6 ± 174.9	224.7 ± 194.5^†^	145.9 ± 169.4
CP volume (mm^3^)	29.6 ± 68.2	67.7 ± 89.9^†^	23.6 ± 62.2
Myocardial morphology			
LV mass (g)	109.6 ± 31.9	107.6 ± 31.2	109.8 ± 32.0
LV mass index (g/m^2^)	56.0 ± 14.6	57.4 ± 15.8	55.8 ± 14.4

*CP*, calcified plaque; *IAP*, intermediate-attenuation plaque; *LAP*, low-attenuation plaque; *LV*, left ventricular

^*^p < 0.05 vs. FFR_CT_ > 0.80. ^†^*p* < 0.01 vs. FFR_CT_ > 0.80

### Effects of RCA branches on FFR_CT_

To investigate the effects of RCA branches on FFR_CT_, changes in FFR_CT_ were compared in the following groups: (1) presence of both right ventricular branch and acute marginal branch confirmed by FFR_CT_ (*n* = 305); (2) presence of right ventricular branch confirmed by FFR_CT_ (*n* = 99); (3) presence of acute marginal branch confirmed by FFR_CT_ (*n* = 28); (4) absence of both right ventricular branch and acute marginal branch confirmed by FFR_CT_ (*n* = 11). Distal FFR_CT_ (group 1, 0.87 ± 0.08; group 2, 0.88 ± 0.08; group 3, 0.88 ± 0.06, and group 4, 0.89 ± 0.03) and ΔFFR_CT_ (group 1, 0.13 ± 0.08; group 2, 0.12 ± 0.08; group 3, 0.12 ± 0.06, and group 4, 0.11 ± 0.03) did not significantly differ between the four groups (Supplementary Table 2).

### Univariate and multivariate analysis of the relationship between FFR_CT_ and vessel morphology

**Fig. 5:**
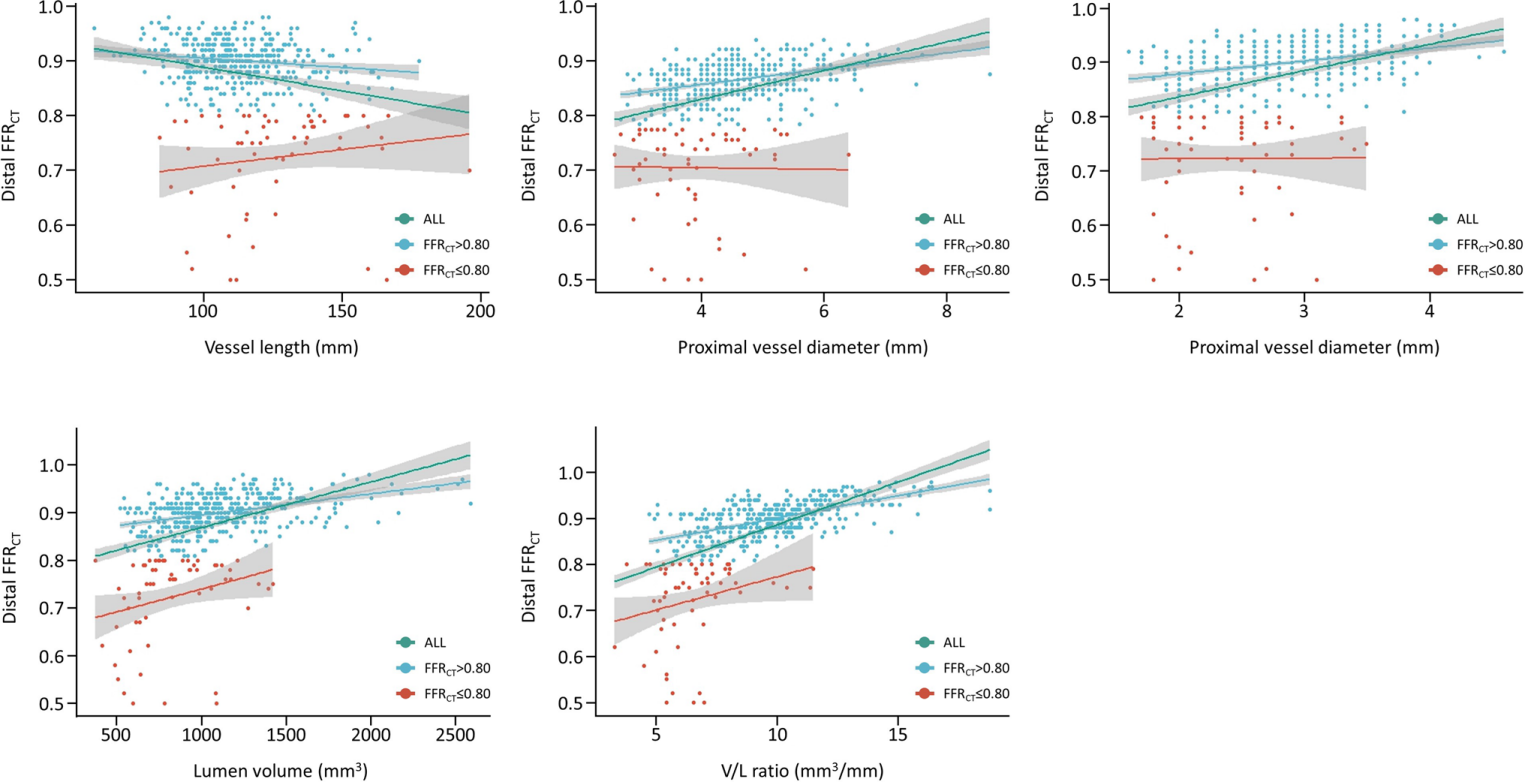
Relationship between distal FFR_CT_ and vessel morphology

**Fig. 6:**
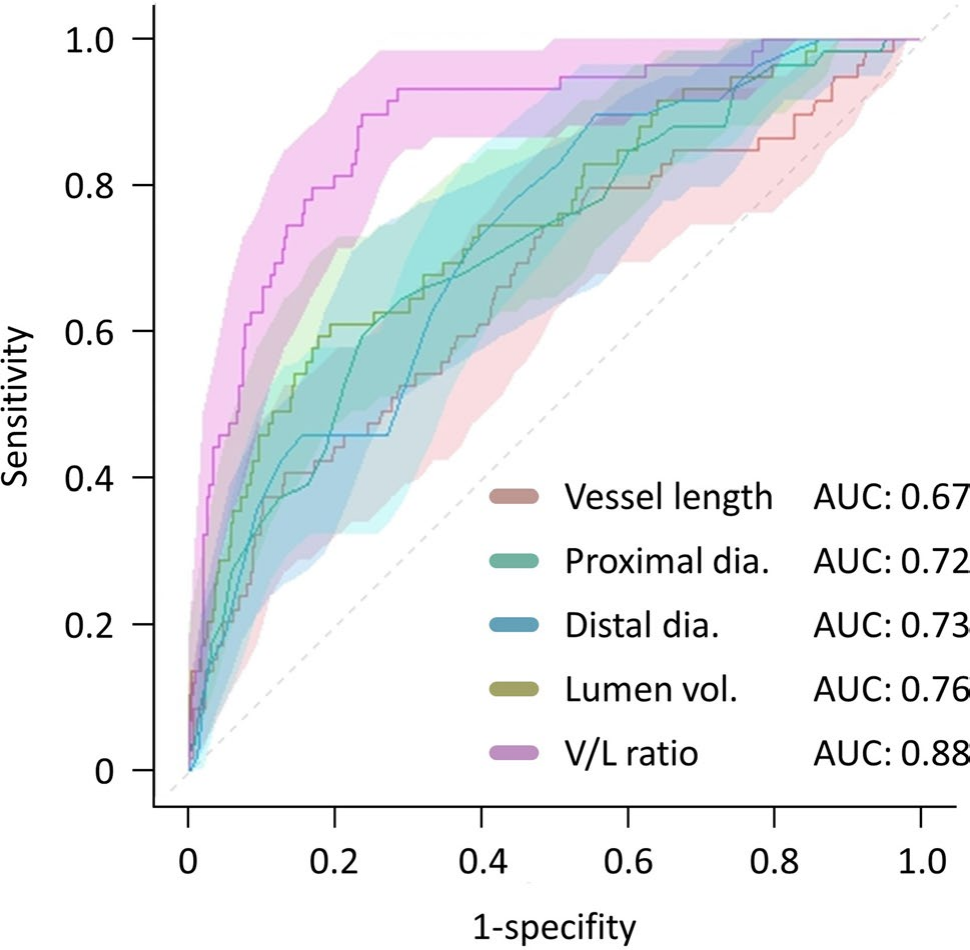
Receiver operating curve of vessel morphology for predicting distal FFR_CT_. dia., diameter; vol., volume

In all patients (FFR_CT_ ≤ 0.80 and > 0.80), distal FFR_CT_ and ΔFFR_CT_ were correlated with vessel-related parameters (proximal and distal vessel diameters, vessel length, lumen diameter, and V/L ratio) and plaque-related parameters (LAP, IAP, and CP volume). Among all vessel-related parameters, V/L ratio showed the highest correlation with distal FFR_CT_ (*r* = 0.61, *p* < 0.0001) (Fig. 5 and Supplementary Table 3) and ΔFFR_CT_ (*r* =  − 0.61, *p* < 0.0001) (Supplementary Fig. 1 and Supplementary Table 4). Multivariable analysis showed that CP volume was the strongest predictor of distal FFR_CT_ (β-coefficient =  − 0.12, *p* = 0.01), followed by V/L ratio (β-coefficient = 0.48, *p* = 0.03) (Table 2). Furthermore, as for ΔFFR_CT_, CP plaque volume (β-coefficient = 0.12, *p* = 0.01) was the strongest predictor, and was followed by V/L ratio (β-coefficient =  − 0.48. *p* = 0.03) (Supplementary Table 5). Among vessel-related parameters, V/L ratio of 8.1 mm^3^/mm had the highest diagnostic performance for an FFR_CT_ at distal RCA ≤ 0.80 (AUC = 0.88, 95% CI 0.84–0.93, *p* < 0.0001, sensitivity 90.0%, specificity 76.7%) (Fig. 6 and Supplementary Table 6).

**Table 2:** Univariable and multivariable analysis for distal FFR_CT_

	Univariable analysis				Multivariable analysis			
	β	95% CI	t-value	*p*-value	β	95% CI	t-value	*p*-value
Vessel length	− 0.22	− 0.001 to − 0.0005	− 4.70	< 0.0001				
Lumen volume	0.42	0.00008 to 0.001	9.82	< 0.0001				
Proximal vessel diameter	0.36	0.02 to 0.04	8.18	< 0.0001	0.09	0.0002 to 0.01	2.02	0.04
Distal vessel diameter	0.37	0.04 to 0.06	8.37	< 0.0001				
V/L ratio	0.61	0.016 to 0.02	16.30	< 0.0001	0.48	0.001 to 0.03	2.14	0.03
LAP volume	− 0.16	− 0.0003 to − 0.04	− 3.30	0.001				
IAP volume	− 0.17	− 0.0001 to 0.00003	− 3.51	0.0005				
CP volume	− 0.19	− 0.0003 to − 0.0001	− 4.09	< 0.0001	− 0.12	− 0.0002 to − 0.00003	− 2.54	0.01
LV mass index	− 0.07	− 0.0009 to 0.0002	− 1.32	0.19				

*CI*, confidence interval; *CP*, calcified plaque; *IAP*, intermediate-attenuation plaque; *LAP*, low-attenuation plaque; *LV*, left ventricular

### Repeatability and reproducibility

Intra-observer and inter-observer variability for vessel-related parameters and myocardium-related parameters is summarised in Supplementary Figs. 2 and 3. Intra-observer and inter-observer analyses showed good repeatability and reproducibility.

## Discussion

In patients with an ICA showing no or minimal CAD in the RCA, our study highlighted the following: (1) vessel length was almost the same in FFR_CT_ ≤ 0.80 and > 0.80, but lumen volume and V/L ratio were significantly lower in the FFR_CT_ ≤ 0.80 group; (2) FFR_CT_ correlated with plaque-related parameters (LAP, IAP, and CP) and vessel-related parameters (proximal and distal vessel diameter, vessel length, lumen volume, and V/L ratio); (3) of all parameters (plaque-related parameters and vessel related-parameters), the strongest predictor of a distal FFR_CT_ value was CP volume followed by V/L ratio and an optimal cut-off value of 8.1 mm^3^/mm. Our study reports for the first time that FFR_CT_ decline depends on V/L ratio in patients with NOCAD in the RCA.

The progressive and continuous FFR_CT_ [3, 4] and invasive FFR [22] decline from the proximal to the distal segments of the vessel even in NOCAD is a commonly observed radiological finding. In our study, 60 patients (14%) with NOCAD had FFR_CT_ < 0.80. Physiological FFR_CT_ decline was dependent on iodine contrast attenuation and the full algorithm remains unclear for commercial reasons. However, FFR_CT_ hemodynamics have been reported to be influenced by various factors The degree of FFR_CT_ decline was not hemodynamically uniform in major three vessels and that RCA was smaller than LAD and LCX. This finding may be related to energy loss due to turbulence by the bifurcation from the left main trunk to LAD and LCX, or splitting into large branches such as diagonal branch, obtuse marginal branch, or posterior descending branch. Our previous study on the ramus artery demonstrated that the presence of a large ramus artery yields energy loss due to turbulence around the bifurcation angle, resulting in distal FFR_CT_ decline in both LAD and LCX [23, 24]. Turbulent eddies generated by the presence of large branches could contribute to energy loss, resulting in impact on FFR_CT_ hemodynamics. RCA may also have side branches such as the right ventricular branch or acute marginal branch (Supplementary Table 2). Turbulence flow generated in the RCA side branches was small and unlikely to affect FFR_CT_. Therefore, RCA with no major branches was included in the present study.

The finding that the degree of FFR_CT_ decline depended on vessel length was consistent with previous reports [3, 4]. Interestingly, even though vessel length was almost the same between FFR_CT_ ≤ 0.80 and > 0.80, proximal/distal vessel diameters, lumen volume, and V/L ratio were significantly lower in FFR_CT_ ≤ 0.80. Vessel morphology including proximal/distal vessel diameters, lumen volume, and V/L ratio may have the potential to play an important role in physiological FFR_CT_ hemodynamics. Several studies have reported the effects of vessel morphology on FFR_CT_ from two perspectives: luminal cross-sectional area (2D) and vessel volume (3D). At the 2D level (luminal cross-sectional area), Collet et al [11] reported that FFR_CT_ was significantly correlated with the luminal cross-sectional area. At the 3D level (lumen volume), Holmes et al [12] reported that increase in homogeneous lumen volume caused by the vasodilator agent of nitroglycerin resulted in FFR_CT_ elevation. According to Hagen-Poiseuille flow, the change in pressure is proportional to the length, and the volumetric flow rate, and inversely proportional to the fourth power of the lumen diameter radius [25]. Baumgartner et al [26] reported that positive remodelling which was heterogeneous vasodilatation was associated with an increase in FFR_CT_. The streamlined vessel morphology caused by positive remodelling leads to pressure recovery phenomena, which may be responsible for the FFR_CT_ elevation. Tapering as a morphological change of the vessel might contribute to FFR_CT_ decline. A small distal vessel diameter was related to FFR_CT_ ≤ 0.80 at the distal segment [9]. Our results of multivariable analysis were consistent with the results of previous studies. Of the vessel morphology-related parameters, V/L ratio was the most predictive factor for distal FFR_CT_. Hence, a thinner and longer vessel contributes to a larger FFR_CT_ decline. Conversely, a shorter and thicker vessel causes a smaller FFR_CT_ decline.

Differences between FFR_CT_ ≤ 0.80 and > 0.80 could be attributed to hemodynamic stress. FFR_CT_ depends on coronary artery flow. Coronary artery flow is determined by hemodynamic stress following three factors: (1) wall shear stress; (2) perfusion pressure; (3) vascular resistance. Of these factors, wall shear stress is affected by vessel diameter (inversely related to vessel diameter). Wall shear stress generates nitric oxide, which increases coronary artery flow, resulting in greater pressure drop and FFR_CT_ decline [27]. In our study, FFR_CT_ ≤ 0.80 group tended to have smaller vessel diameters than > 0.80, which might contribute to high shear stress and small FFR_CT_ values. To our knowledge, this is the first report to investigate the effect of the ratio of lumen volume to vascular length on FFR_CT_. The addition of a novel parameter, V/L ratio assessment in addition to the conventional FFR_CT_ interpretation may have the potential to overcome some drawbacks of FFR_CT_. Decision making based on FFR_CT_ values at distal coronary artery segments should be performed with the greatest caution and by a thorough consideration of lumen volume.

## Limitations

Our study has some limitations. First, invasive FFR was not performed because included patients were NOCAD. Since FFR_CT_ is highly concordant with invasive FFR, it can be used as an alternative testing method [28–30]. Our study excluded a history of old myocardial infarction, but may not have completely ruled out the effects of left coronary artery disease. Old myocardial infarction causes left ventricular (LV) dysfunction, resulting in reduced left ventricular myocardial–related parameters such as LV wall thickness or LV mass. Fairbairn et al reported that the ratio of lumen volume to LV mass (V/M ratio) and FFR_CT_ were inversely related. In our previous study, LV mass index was the most influential factor on FFR_CT_ among LV myocardial-related parameters, with a cut-off value of 66.5 g/m^2^. Furthermore, LV dysfunction due to old myocardial infarction or heart failure causes an increase in LV end-diastolic pressure or low cardiac output, resulting in a decrease in right ventricular perfusion. In the setting of low right ventricular perfusion, the pressure drop is lower than normally expected coronary blood flow, resulting in higher FFR_CT_ values. Collateral circulation generates unusual coronary hemodynamics from donor artery perfusion. FFR_CT_ values depend on the donor and recipient arteries balance (Supplementary Fig. 4). In our study, patients with old myocardial infarction or heart failure were excluded, and it is unlikely that right ventricular perfusion would be compromised due to low cardiac output. LV mass index was below the threshold to affect FFR_CT_ hemodynamics and was not a strong predictor of distal FFR_CT_ and Δ FFR_CT_ in multivariate analysis. Furthermore, there was no evidence of collateral circulation. Therefore, left coronary artery disease was unlikely to affect FFR_CT_ in the RCA. Second, this study focused on only RCA vessels to avoid the effects of energy loss due to the bifurcation branches. Our results should be confirmed with all vessels including left coronary arteries. Third, despite the fact that FFR_CT_ is affected by plaque volume [3, 31–33], this study did not completely eliminate plaque volume even though only NOCAD were selected. However, it is controversial to investigate FFR_CT_ in simulated vessel models that completely eliminates the effects of plaque characteristics. Simulated vessel models are assumed to have a rigid wall rather than an elastic wall; therefore, the simulation does not reflect the physiological situation [34].

## Conclusions

A novel marker for the predictor of distal FFR_CT_, V/L ratio is useful in assessing the physiological changes in subclinical perfusion disturbance in coronary arteries.

### Supplementary Information

Below is the link to the electronic supplementary material.

Supplementary file1 (3.10 MB)

## Abbreviations

CAD: Coronary artery disease
CAD-RADS: CAD reporting and data system
CP: Calcified plaque
CTA: CT angiography
FFR: Fractional flow reserve
FFR_CT_: Computed tomography–derived fractional flow reserve
HU: Hounsfield units
IAP: Intermediate-attenuation plaque
ICA: Invasive coronary angiography
LAD: Left anterior descending artery
LAP: Low-attenuation plaque
LCX: Left circumflex artery
LV: Left ventricular
NOCAD: Non-obstructive CAD
RCA: Right coronary artery

## Declarations

### Guarantor

The scientific guarantor of this publication is Johan De Mey.

### Statistics and biometry

No complex statistical methods were necessary for this paper.

### Methodology

- retrospective
- observational
- performed at one institution
