# Supplementary material for: Impact of vessel morphology on CT-derived fractional-flow-reserve in non-obstructive coronary artery disease in right coronary artery
Source: Eur Radiol. 2023 Sep 1;34(3):1836–45. doi: 10.1007/s00330-023-09972-8 (PMC10873436; doi:10.1007/s00330-023-09972-8)
Supplement: Supplementary file 1 — Supplementary file1 (3.10 MB) [file 330_2023_9972_MOESM1_ESM.pdf]

Supplementary Table 1. FFR_{CT} changes in each right coronary artery segment

	Overall<br>n =443	95% CI<br>n =443	$FFR_{CT} \leq 0.80$<br>n = 60	95% CI<br>n = 60	$FFR_{CT} > 0.80$<br>n = 383	95% CI<br>n = 383
#1 Proximal	1.00 ± 0.00	1.00 to 1.00	1.00 ± 0.00	1.00 to 1.00	1.00 ± 0.00	1.00 to 1.00
#1 Middle	0.98 ± 0.02	0.98 to 0.98	0.97 ± 0.03*	0.96 to 0.98	0.98 ± 0.02	0.98 to 0.98
#1 Distal	0.96 ± 0.03	0.96 to 0.96	0.93 ± 0.06†	0.91 to 0.94	0.96 ± 0.02	0.96 to 0.97
#2 Proximal	0.94 ± 0.05	0.94 to 0.95	0.88 ± 0.10†	0.86 to 0.91	0.95 ± 0.03	0.95 to 0.95
#2 Middle	0.93 ± 0.06	0.92 to 0.93	0.84 ± 0.10†	0.81 to 0.86	0.94 ± 0.03	0.95 to 0.95
#2 Distal	0.92 ± 0.06	0.91 to 0.92	0.81 ± 0.10†	0.79 to 0.84	0.94 ± 0.03	0.93 to 0.94
#3 Proximal	0.91 ± 0.07	0.90 to 0.91	0.78 ± 0.10†	0.75 to 0.80	0.93 ± 0.03	0.92 to 0.93
#3 Middle	0.89 ± 0.07	0.89 to 0.90	0.75 ± 0.09†	0.73 to 0.78	0.91 ± 0.04	0.91 to 0.92
#3 Distal	0.88 ± 0.08	0.87 to 0.89	0.72 ± 0.09†	0.70 to 0.75	0.99 ± 0.04	0.90 to 0.90
ΔFFR_{CT}	0.12 ± 0.08	0.12 to 0.13	0.28 ± 0.09†	0.25 to 0.30	0.10 ± 0.04	0.10 to 0.10

*P < 0.05 vs. $FFR_{CT} > 0.80$. †P < 0.01 vs. $FFR_{CT} > 0.80$.

Supplementary Table 2. Effects of RCA branches on FFR_{CT}

	**Group 1 Presence of RVB and AM (n = 305)**	**Group 2 Presence of RVB (n = 99)**	**Group 3 Presence of AM (n = 28)**	**Group 4 Absence of RVB and AM (n = 11)**
**FFR_{CT} characteristics**				
Proximal FFR_{CT}	1.00 ± 0.00	1.00 ± 0.00	1.00 ± 0.00	1.00 ± 0.00
Distal FFR_{CT}	0.87 ± 0.08	0.88 ± 0.08	0.88 ± 0.06	0.89 ± 0.03
ΔFFR_{CT}	0.13 ± 0.08	0.12 ± 0.08	0.12 ± 0.06	0.11 ± 0.03
**Vessel morphology**				
Proximal vessel diameter (mm)	4.6 ± 1.0	4.5 ± 1.0	4.5 ± 1.0	4.4 ± 1.1
Distal vessel diameter (mm)	2.8 ± 0.6	2.9 ± 0.6	2.8 ± 0.5	2.9 ± 0.6
Vessel length (mm)	115.6 ± 20.2	110.7 ± 17.4	120.4 ± 19.5	112.5 ± 9.4
Lumen volume (mm^3)	1072.7 ± 347.8	1058.8 ± 349.4	1143.8 ± 267.9	1070.6 ± 320.3
V/L ratio (mm^3/mm)	9.3 ± 2.6	9.6 ± 2.6	9.6 ± 2.3	9.5 ± 2.2
Low attenuation plaque volume (mm^3)	15.8 ± 19.5	15.0 ± 16.4	15.5 ± 18.2	16.3 ± 11.9
Intermediate attenuation plaque volume (mm^3)	153.2 ± 158.6	153.1 ± 172.7	210.6 ± 321.5	144.9 ± 83.3
Calcified plaque volume (mm^3)	31.7 ± 74.3	28.6 ± 59.0	17.3 ± 30.7	11.6 ± 18.7

No significant difference between each group.

Supplementary Table 3. Relationship between distal FFR_{CT} vessel parameters.

	ALL n = 443			$FFR_{CT} \leq 0.80$ n = 60			$FFR_{CT} > 0.80$ n = 383		
	R	95% CI	P-value	R	95% CI	P-value	R	95% CI	P-value
Vessel length	−0.22	−0.3–−0.1	<0.0001	0.16	−0.1–0.4	0.2	−0.16	−0.3–−0.06	0.001
Lumen volume	0.42	0.3–0.5	<0.0001	0.27	0.02–0.5	0.04	0.40	0.3–0.5	<0.0001
Proximal vessel diameter	0.36	0.3–0.4	<0.0001	−0.01	−0.3–−0.2	0.9	0.41	0.3–0.5	<0.0001
Distal vessel diameter	0.37	0.3–0.5	<0.0001	0.005	−0.2–−0.3	1.0	0.37	0.3–0.5	<0.0001
V/L ratio	0.61	0.6–0.7	<0.0001	0.26	0.005–0.5	0.046	0.61	0.5–0.7	<0.0001
LAP volume	−0.16	−0.3–−0.06	0.001	0.07	−0.2–0.3	0.6	−0.28	−0.4–−0.2	<0.0001
IAP volume	−0.17	−0.3–−0.07	0.0005	0.08	−0.2–0.3	0.5	−0.15	−0.22–−0.05	0.004
CP volume	−0.19	−0.3–−0.1	<0.0001	0.05	−0.2–0.3	0.7	−0.05	−0.2–−0.04	0.3
LV mass index	−0.07	−0.2–−0.03	0.2	−0.05	−0.3–0.22	0.7	−0.12	−0.2–−0.01	0.03

CI, confidence interval; CP, calcified plaque; IAP, intermediate-attenuation plaque; LAP, low-attenuation plaque; LV, left ventricular.

Supplementary Table 4. Relationship between ΔFFR_{CT} and vessel parameters.

	ALL n = 443			$FFR_{CT} \leq 0.80$ n = 60			$FFR_{CT} > 0.80$ n = 383		
	R	95% CI	P-value	R	95% CI	P-value	R	95% CI	P-value
Vessel length	0.22	0.1–0.3	<0.0001	− 0.16	− 0.4–0.11	0.2	0.16	0.06–0.3	0.002
Lumen volume	− 0.43	− 0.5– − 0.3	<0.0001	− 0.27	− 0.5– − 0.02	0.04	− 0.41	− 0.5– − 0.3	<0.0001
Proximal vessel diameter	− 0.36	− 0.4– − 0.3	<0.0001	0.01	− 0.1–0.3	0.9	− 0.41	− 0.5– − 0.3	<0.0001
Distal vessel diameter	− 0.37	− 0.4– − 0.3	<0.0001	− 0.006	− 0.4–0.2	1.0	− 0.37	− 0.5– − 0.3	<0.0001
V/L ratio	− 0.61	− 0.77– − 0.6	<0.0001	− 0.26	− 0.5– − 0.005	0.046	− 0.61	− 0.77– − 0.5	<0.0001
LAP volume	0.16	0.06–0.2	0.001	− 0.07	− 0.3–0.2	0.6	0.28	0.2–0.4	<0.0001
IAP volume	0.17	0.07–0.3	0.0005	− 0.099	− 0.30.2	0.5	− 0.15	0.05–0.2	0.004
CP volume	0.19	0.1–0.3	<0.0001	− 0.02	− 0.3–0.2	0.9	0.06	− 0.04–0.2	− 0.2

LV mass index	0.07	− 0.03–0.2	0.2	− 0.05	− 0.3–0.2	0.7	0.12	0.01–0.2	0.03

CI, confidence interval; CP, calcified plaque; IAP, intermediate-attenuation plaque; LAP, low-attenuation plaque; LV, left ventricular.

Supplementary Table 5. Univariable and multivariable analysis for ΔFFR$_{CT}$

	Univariable analysis				Multivariable analysis			
	**β**	**95% CI**	**t-value**	***P*-value**	**β**	**95% CI**	**t-value**	***P*-value**
Vessel length	0.22	0.0005 to 0.001	4.67	<0.0001				
Lumen volume	-0.43	-0.0001 to -0.00008	-9.86	<0.0001				
Proximal vessel diameter	-0.36	-0.04 to -0.02	-8.16	<0.0001	-0.09	-0.01 to -0.0001	-2.00	0.046
Distal vessel diameter	-0.37	-0.06 to -0.04	-8.38	<0.0001				
V/L ratio	-0.61	-0.02 to -0.016	-16.33	<0.0001	-0.48	-0.03 to -0.001	-2.18	0.03
LAP volume	0.16	0.0003 to 0.001	3.30	0.001				
IAP volume	0.17	0.00003 to 0.0001	3.52	0.0005				
CP volume	0.19	0.0001 to 0.0003	4.11	<0.0001	0.12	0.00003 to 0.0002	2.56	0.01
LV mass index	0.07	-0.0002 to 0.0009	1.32	0.19				

CI, confidence interval; CP, calcified plaque; IAP, intermediate-attenuation plaque; LAP, low-attenuation plaque; LV, left ventricular.

Supplementary Table 6. Receiver operating curve of vessel morphology for predicting distal FFR_{CT}.

	Cut-off	AUC	95% CI	Sensitivity	Specificity	P-value
Vessel length (mm)	132.1	0.67	0.59–0.75	40.0	86.9	< 0.0001
Proximal vessel diameter (mm)	3.9	0.72	0.65–0.79	60.0	76.7	< 0.0001
Distal vessel diameter (mm)	2.9	0.73	0.67–0.80	90.0	44.5	< 0.0001
Lumen volume (mm^3)	846.7	0.76	0.69–0.83	61.7	80.9	< 0.0001
V/L ratio (mm^3/mm)	8.1	0.88	0.84–0.93	90.0	76.7	< 0.0001

AUX; area under the curve, CI, confidence interval.

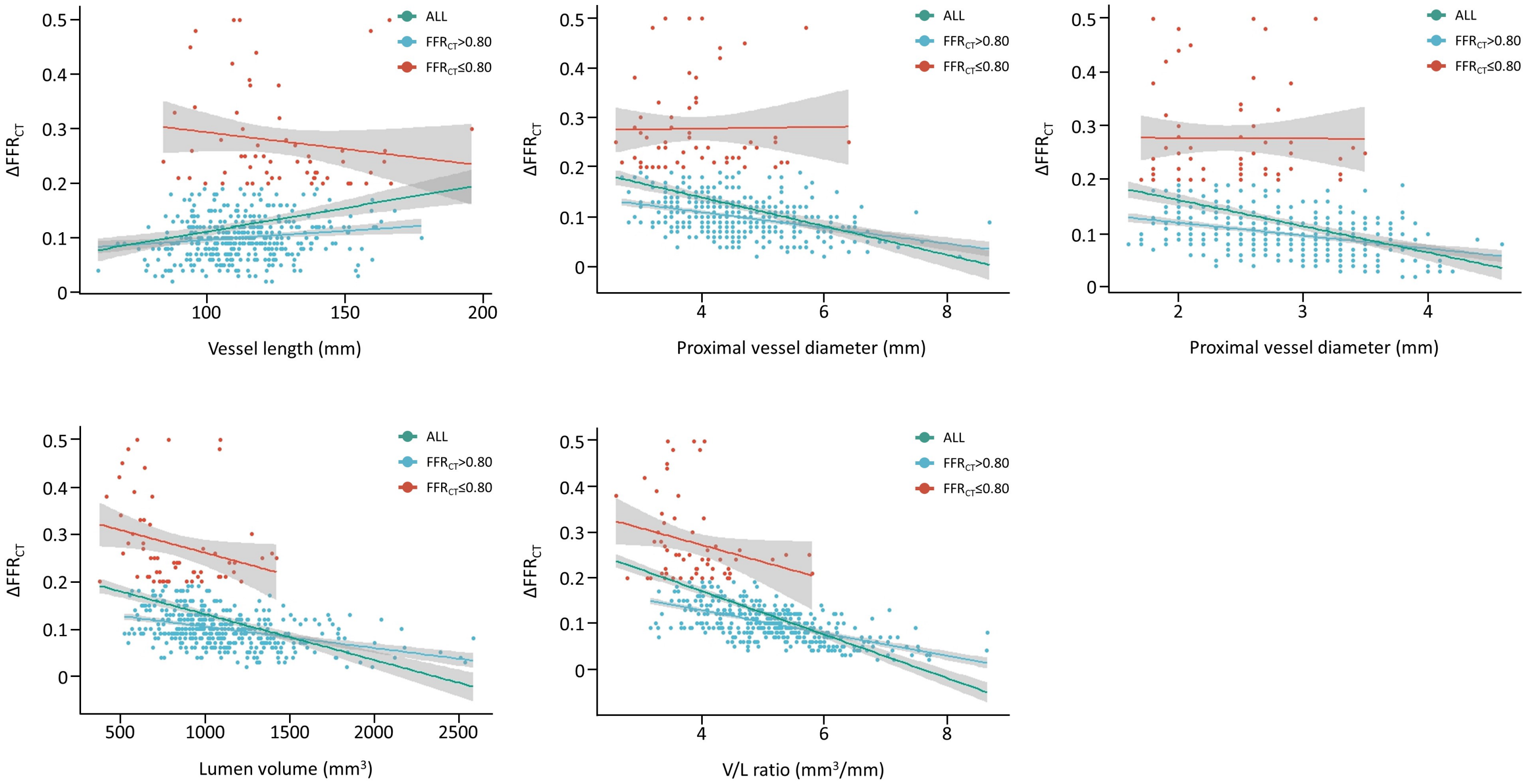
ΔFFRCT
0.5
0.4
0.3
0.2
0.1
0
ALL
FFRCT>0.80
FFRCT≤0.80
100
150
200
Vessel length (mm)
4
6
8
Proximal vessel diameter (mm)
2
3
4
Proximal vessel diameter (mm)
500
1000
1500
2000
2500
Lumen volume (mm3)
4
6
8
V/L ratio (mm3/mm)

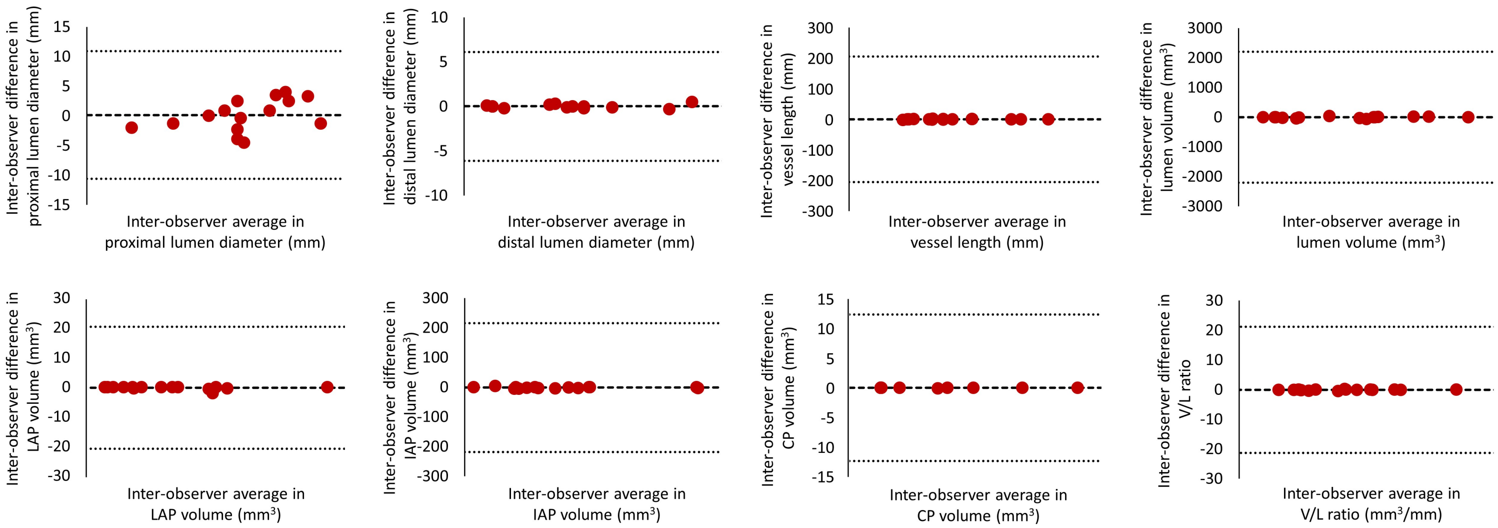
Inter-observer difference in proximal lumen diameter (mm)
Inter-observer average in proximal lumen diameter (mm)
Inter-observer difference in distal lumen diameter (mm)
Inter-observer average in distal lumen diameter (mm)
Inter-observer difference in vessel length (mm)
Inter-observer average in vessel length (mm)
Inter-observer difference in lumen volume (mm3)
Inter-observer average in lumen volume (mm3)
Inter-observer difference in LAP volume (mm3)
Inter-observer average in LAP volume (mm3)
Inter-observer difference in IAP volume (mm3)
Inter-observer average in IAP volume (mm3)
Inter-observer difference in CP volume (mm3)
Inter-observer average in CP volume (mm3)
Inter-observer differencie in V/L ratio
Inter-observer average in V/L ratio (mm3/mm)

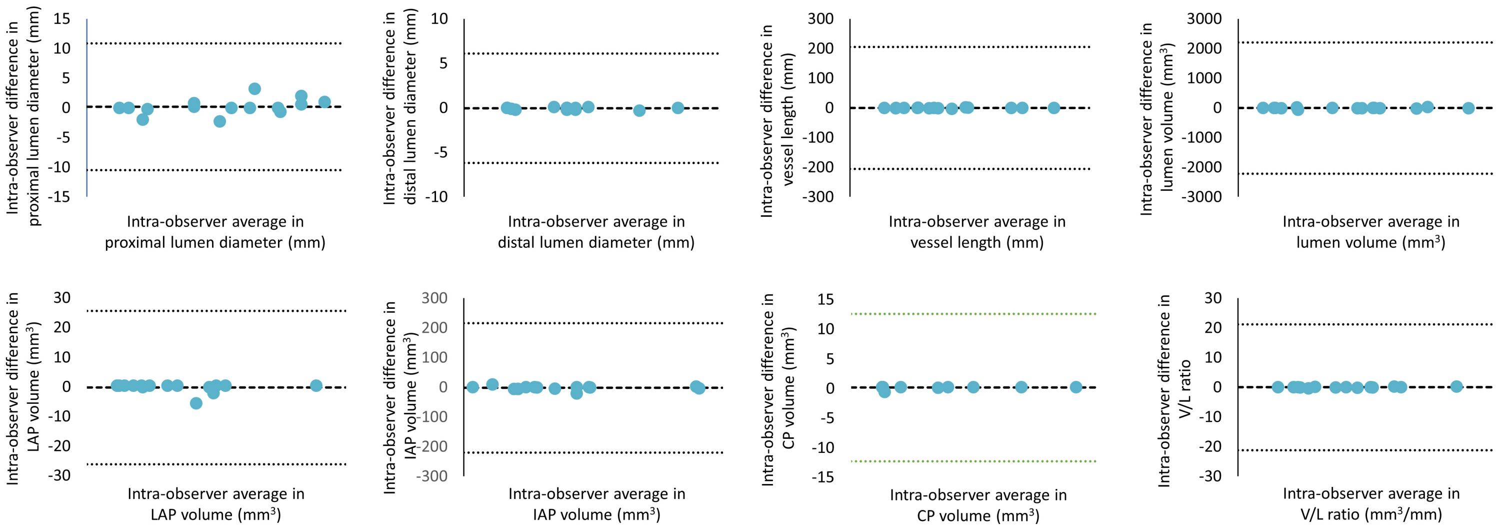
Intra-observer difference in proximal lumen diameter (mm)
15
10
5
0
-5
-10
-15
Intra-observer average in proximal lumen diameter (mm)
Intra-observer difference in distal lumen diameter (mm)
10
5
0
-5
-10
Intra-observer average in distal lumen diameter (mm)
Intra-observer difference in vessel length (mm)
300
200
100
0
-100
-200
-300
Intra-observer average in vessel length (mm)
Intra-observer difference in lumen volume (mm3)
3000
2000
1000
0
-1000
-2000
-3000
Intra-observer average in lumen volume (mm3)
Intra-observer difference in LAP volume (mm3)
30
20
10
0
-10
-20
-30
Intra-observer average in LAP volume (mm3)
Intra-observer difference in IAP volume (mm3)
300
200
100
0
-100
-200
-300
Intra-observer average in IAP volume (mm3)
Intra-observer difference in CP volume (mm3)
15
10
5
0
-5
-10
-15
Intra-observer average in CP volume (mm3)
Intra-observer difference in V/L ratio
30
20
10
0
-10
-20
-30
Intra-observer average in V/L ratio (mm3/mm)

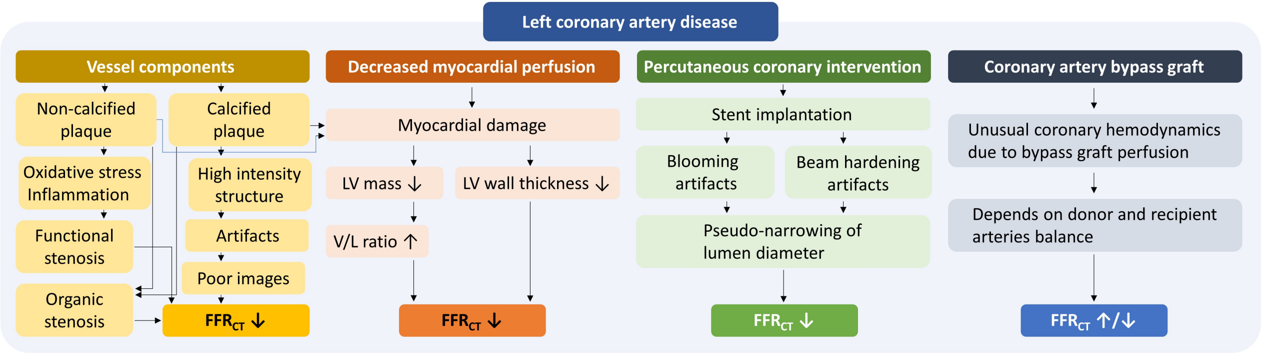
Left coronary artery disease
Vessel components
Non-calcified plaque
Calcified plaque
Oxidative stress Inflammation
High intensity structure
Functional stenosis
Artifacts
Poor images
Organic stenosis
FFR$_{CT}$ ↓
Decreased myocardial perfusion
Myocardial damage
LV mass ↓
LV wall thickness ↓
V/L ratio ↑
FFR$_{CT}$ ↓
Percutaneous coronary intervention
Stent implantation
Blooming artifacts
Beam hardening artifacts
Pseudo-narrowing of lumen diameter
FFR$_{CT}$ ↓
Coronary artery bypass graft
Unusual coronary hemodynamics due to bypass graft perfusion
Depends on donor and recipient arteries balance
FFR$_{CT}$ ↑/↓
